# Systematic Review and Meta-Analysis of the Efficacy and Safety of Existing TNF Blocking Agents in Treatment of Rheumatoid Arthritis

**DOI:** 10.1371/journal.pone.0030275

**Published:** 2012-01-17

**Authors:** Kalle J. Aaltonen, Liisa M. Virkki, Antti Malmivaara, Yrjö T. Konttinen, Dan C. Nordström, Marja Blom

**Affiliations:** 1 Faculties of Pharmacy and Medicine, University of Helsinki, Helsinki, Finland; 2 Faculty of Medicine, University of Helsinki, Helsinki, Finland; 3 Centre for Health and Social Economics, National Institute for Health and Welfare (THL), Helsinki, Finland; 4 COXA Hospital for Joint Replacement, Tampere, Finland; 5 Helsinki University Central Hospital (HUCH), Helsinki, Finland; 6 Faculty of Pharmacy, University of Helsinki, Helsinki, Finland; Lerner Research Institute, Cleveland Clinic, United States of America

## Abstract

**Background and Objectives:**

Five-tumour necrosis factor (TNF)-blockers (infliximab, etanercept, adalimumab, certolizumab pegol and golimumab) are available for treatment of rheumatoid arthritis. Only few clinical trials compare one TNF-blocker to another. Hence, a systematic review is required to indirectly compare the substances. The aim of our study is to estimate the efficacy and the safety of TNF-blockers in the treatment of rheumatoid arthritis (RA) and indirectly compare all five currently available blockers by combining the results from included randomized clinical trials (RCT).

**Methods:**

A systematic literature review was conducted using databases including: MEDLINE, SCOPUS (including EMBASE), Cochrane library and electronic search alerts. Only articles reporting double-blind RCTs of TNF-blockers *vs.* placebo, with or without concomitant methotrexate (MTX), in treatment of RA were selected. Data collected were information of patients, interventions, controls, outcomes, study methods and eventual sources of bias.

**Results:**

Forty-one articles reporting on 26 RCTs were included in the systematic review and meta-analysis. Five RCTs studied infliximab, seven etanercept, eight adalimumab, three golimumab and three certolizumab. TNF-blockers were more efficacious than placebo at all time points but were comparable to MTX. TNF-blocker and MTX combination was superior to either MTX or TNF-blocker alone. Increasing doses did not improve the efficacy. TNF-blockers were relatively safe compared to either MTX or placebo.

**Conclusions:**

No single substance clearly rose above others in efficacy, but the results of the safety analyses suggest that etanercept might be the safest alternative. Interestingly, MTX performs nearly identically considering both efficacy and safety aspects with a margin of costs.

## Introduction

Rheumatoid arthritis (RA) is an inflammatory autoimmune disease with a prevalence of 0.5–1.0 per cent in Northern Europe [1]. A recent epidemiological study from Sweden reported that 0.77% of the population have been diagnosed with RA while a survey from UK found the prevalence to be 0.82% [2], [3]. RA is usually diagnosed before the age of 60 and is more common in women than men. Both genetic and environmental factors play a role [4]. Symptoms include joint destruction, pain and impaired movement.

Since the discovery of the role of tumour necrosis factor (TNF) in chronic inflammation in RA, five drugs based on blocking TNF have entered clinical use. Infliximab, adalimumab, golimumab and certolizumab pegol (certolizumab) are monoclonal antibodies targeted against TNF whereas etanercept is a soluble TNF-receptor [5]. However, only few clinical trials compared one TNF-blocker to other TNF-blockers. Previous systematic reviews and meta-analyses have studied the subject in various settings and comparisons [6]–[14]. These studies concluded that while TNF-blockers are efficacious but it may still be beneficial to use them in combination therapies. Only few differences in efficacy and safety between individual substances were discovered. However, more randomized clinical trials have been published lately with additional data available to systematic reviews and most importantly, two new substances, certolizumab and golimumab, have been introduced to clinical use.

The purpose of this systematic review and meta-analysis is to study the efficacy and safety of all five currently available TNF-blockers in the treatment of RA compared to either methotrexate (MTX) and placebo or placebo alone and to perform an indirect comparison between individual substances in different drug combinations and doses and at different time points. We test the assumption that it is more efficacious and comparatively safer to use MTX in combination with a TNF-blocker in the treatment of RA compared to TNF-blocker monotherapy. We study if high doses of TNF-blockers differ from regular doses in efficacy and safety. Primary efficacy endpoint is the risk ratio between intervention and control group in American College of Rheumatology (ACR) 50% improvement at 6 months [15], [16]. Secondary efficacy endpoints include risk ratios in ACR 20%, 50% and 70% improvements at 3, 6 and 12 months in several comparisons. Primary safety endpoint is the risk ratio between intervention and control group in the number of discontinuations due to adverse events. Secondary safety endpoints include risk ratios in the number of adverse events, serious adverse events, infections, serious infections and injection site reactions.

## Methods

### Study selection criteria

We performed a search for randomized clinical trials of five TNF-blockers in treatment of RA. Systematic review was conducted in accordance to methods and recommendations from the Cochrane handbook [17].

According to inclusion criteria patients had to be at least 16 years of age; be diagnosed with RA using ACR 1987 criteria; and be randomized either to intervention or control group. Studies were to have one (or more) of the TNF-blockers as intervention and either placebo or combination of placebo and methotrexate as control. The TNF-blocker had to be delivered through the same route as the commercial drug and be within the dose range recommended for the commercially available products. Efficacy was measured in terms of ACR 20%, 50% and 70% improvements and thus, at least one of these had to be reported at some time point. Information regarding safety had to be reported. Previously published systematic reviews were searched for, but excluded from the systematic review due to the inclusion criteria. The protocol of the study was not published online.

### Search strategy

Search strategy was designed and performed by a librarian by our request. We used the search terms rheumatoid arthritis, anti-TNF, infliximab, etanercept, adalimumab, golimumab, certolizumab, randomized clinical trials and systematic review. Variations in spelling were taken into account. References from (Ovid^®^) Medline, Cochrane library (Cochrane Central register of Controlled Trials, Cochrane Database for Systematic Reviews, Health Technology Assessment, Database of Abstracts of Reviews of Effects, NHS Economic Evaluation, Cochrane Methodology Register), SCOPUS (including Embase), ISI web of knowledge and several other databases were extracted and imported to reference management software (RefWorks). Clinical trial register (clinicaltrials.gov) was hand searched for unpublished trials. Duplicate entries were removed using an automated feature. There were no restrictions on study language. For search strategy, see table S1.

### Study selection

References were evaluated by two individual investigators (KA, LV) using pre-defined inclusion and exclusion criteria. Decision for inclusion was made on consensus. A third investigator (YTK) made the final decision in case of disagreement. Evaluation was based on title and abstract whenever available. Full text articles from potentially relevant references were obtained in electronic or printed format and re-evaluated for inclusion by the same investigators as before. The acronym PICOS (patients, interventions, comparators, outcomes and settings) was used to assess if the references fully complied with the inclusion and exclusion criteria. As full-text article was required for the systematic review and meta-analysis, references whose full-texts we could not acquire either electronically or as printed copies from the University of Helsinki medical library were excluded. Multiple reports from a single study were considered as one study.

### Evaluation for bias

As instructed in the Cochrane handbook for systematic reviews of interventions, the investigators performed an evaluation of bias rather than of methodological quality. Studies included were evaluated for an eventual bias using methods described in the Cochrane handbook. The study was to be considered “possibly biased” in case a possible source of bias was found in any of the seven dimensions evaluated. The following dimensions were considered in the bias assessment tool: Allocation sequence generation, allocation concealment, blinding of participants, personnel and outcome, incomplete outcome data, selective outcome reporting and other sources of bias. Evaluation was done by two independent assessors (KA, LV) to improve the validity. The effect of possible bias on results was studied by performing all meta-analysis twice with possibly biased RCTs included and excluded.

### Data extraction

Data on study design, patient status and background, efficacy and safety were extracted from the publications using an Excel data extraction form by two independent researchers (KA, LV) to improve validity.

### Meta-analyses

Data were analyzed using the intention to treat results from the included studies. Meta-analyses were performed using Cochrane Collaboration Review Manager 5.0 software. Sensitivity analyses were employed to account for the possible bias. In some settings several time points were combined to increase the power. Efficacy and safety were analyzed using dichotomous data to obtain risk ratios. Dichotomous efficacy data included ACR 20%, 50% and 70% improvements whereas dichotomous safety data was composed of the proportion of patients who experienced an adverse outcome or discontinued the treatment due to adverse events. The efficacy and safety of TNF-blockers was analyzed in six different main comparisons. Random effects model was used to account for the diversity of the studies. Heterogeneity was evaluated via subgroup analysis using Chi square and I^2^-statistics.

## Results

### Search results

5308 references were identified from electronic databases by a systematic literature search performed 5.-26.2.2010. 1613 were identified as duplicates by an automated feature in RefWorks. Additionally, 146 references were added via “search alerts”, which extended time coverage of the search to 30.6.2010. No additional references were identified from alternative sources including clinical trial registers.

### Study selection

**Figure 1:**
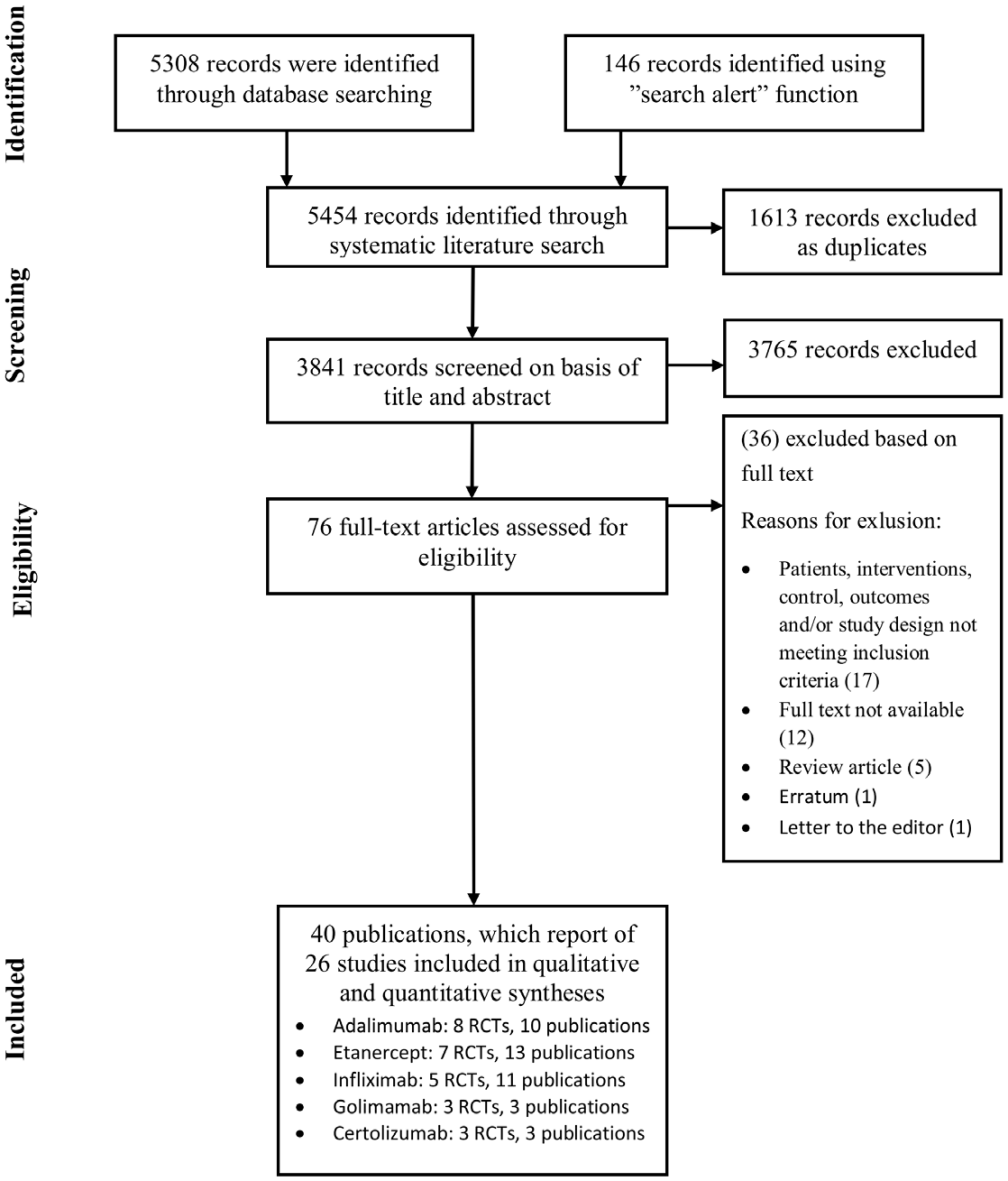
Flowchart of the selection process of the RCTs for the systematic review and meta-analysis.

After removing duplicate entries, 3841 references were evaluated for inclusion based on title and/or abstract. Seventy six potentially relevant references were included in the next stage, where the publication was to be re-evaluated based on full text (figure 1). Full text was unavailable for 12 studies most of which were conference abstracts identified from ISI Web of Knowledge [18]–[29]. Patients, interventions, controls, outcomes or design of the studies did not meet the inclusion criteria of the systematic review in 17 publications [30]–[46]. Five review articles, one letter to the editor [47] and one erratum [48] were excluded. Several of the remaining 40 publications were reporting on a single study and were thus merged into one (table S2). Publications included in the systematic review and meta-analysis are listed in the bibliography with numbers 49–88. From the 26 clinical trials included in the systematic review, 8 used adalimumab, 7 etanercept , 5 infliximab, 3 golimumab and 3 certolizumab for intervention. The included trials have 9862 patients of which 6780 and 3082 were in intervention and control groups, respectively (table S2).

### Evaluation for bias

A potential source of bias was discovered in five trials included in the systematic review (table 1). In many clinical trials there was an early escape route for patients with insufficient treatment response to avoid rapid disease progression. In some studies this was implemented by considering all patients failing to meet a pre-defined treatment response criteria (e.g. ACR 20% improvement) as “non-responders” before the actual efficacy assessment. While this may be for the best interest of the study subjects, it may introduce a bias to the evaluation of the efficacy results. Another bias was caused by switching the control group to active medication.

**Table 1:** The results of an assessment for bias in accordance to a tool by Cochrane Collaboration.*

Study	*Sequence Generation*	*Allocation Concealment*	*Blinding*	*Incomplete Outcome Data*	*Selective Outcome Reporting*	*Other Potential Threats To Validity*
**Infliximab**						
Abe 2006	Unclear	Unclear	Yes	Yes	Yes	Yes
Maini 1999	Yes	Yes	Yes	Yes	Yes	Yes
Quinn 2005	Unclear	Unclear	Yes	Unclear	Yes	Unclear
St. Clair 2004	Yes	Yes	Yes	Unclear	Yes	Yes
Schiff 2008	Unclear	Unclear	Yes	Yes	Yes	Yes until 6 mo/No
**Etanercept**						
Bathon 2000	Unclear	Unclear	Yes	Unclear	Yes	Yes
Emery 2008	Yes	Yes	Yes	Unclear	Yes	Yes
Keystone 2004	Unclear	Unclear	Yes	Yes	Yes	Yes until 8 wk/No
Klareskog 2004	Yes	Yes	Yes	Yes	Yes	Yes
Lan 2004	Unclear	Unclear	Yes	Yes	Yes	Yes
Moreland 1999	Unclear	Yes	Yes	Yes	Yes	Yes
Weinblatt 1999	Unclear	Yes	Yes	Yes	Yes	Yes
**Adalimumab**						
Breedveld 2006	Unclear	Unclear	Yes	Yes	Yes	Yes
Chen 2009	Unclear	Unclear	Yes	Yes	Yes	Yes
Keystone 2004	Unclear	Unclear	Yes	Yes	Yes	Yes
Kim 2007	Unclear	Unclear	Yes	Yes	Yes	Yes
Miyasaka 2008	Unclear	Unclear	Yes	Yes	Yes	No
Van de Putte 2003	Unclear	Unclear	Yes	Yes	Yes	Yes
Van de Putte 2004	Yes	Yes	Yes	Yes	Yes	Yes
Weinblatt 2003	Unclear	Unclear	Yes	Yes	Yes	Yes
**Golimumab**						
Emery 2009	Yes	Yes	Yes	Yes	Yes	Yes
Kay 2008	Unclear	Unclear	Yes	Yes	Yes	Yes
Keystone 2009	Yes	Yes	Yes	Unclear	Yes	Yes until 16 wk/No
**Certolizumab**						
Fleischmann 2008	Yes	Yes	Yes	Yes	Yes	Yes
Keystone 2008	Unclear	Unclear	Yes	Yes	Yes	No
Smolen 2009	Unclear	Unclear	Yes	Yes	Yes	No

*Yes = free of bias, No = possible source of bias, Unclear = not enough information to make the decision.

### Efficacy

#### TNF-blocker vs. control

**Figure 2:**
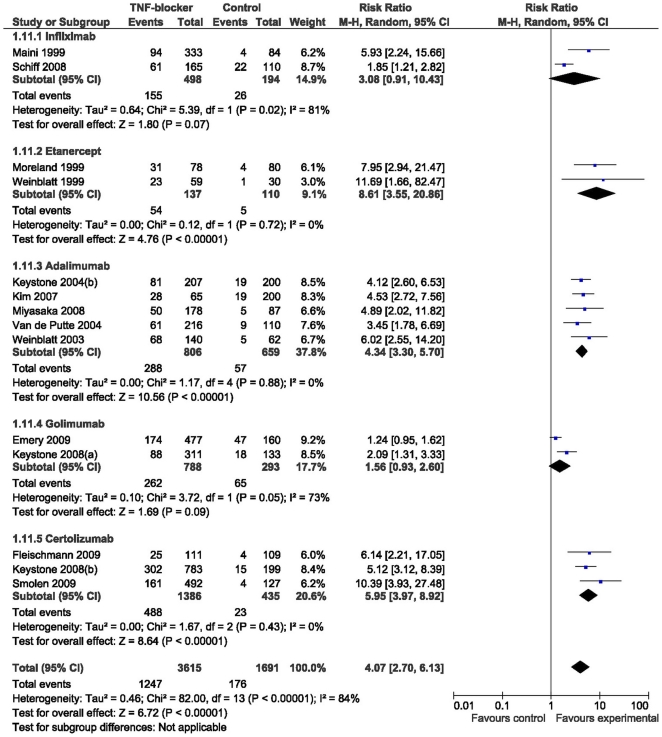
Forest plot of the ACR 50 response at 6 months.

The primary efficacy endpoint of our study was the risk ratio of 50% improvements in the ACR-treatment response criteria at six months between intervention and control group. Fourteen trials were included and of them 2 used infliximab, 2 etanercept, 5 adalimumab, 2 golimumab and 3 certolizumab for intervention. As a group, TNF-blockers reached a risk ratio of 4.07 (95% CI 2.70–6.13) regarding the achievement of the efficacy endpoint compared to controls. For infliximab, etanercept, adalimumab, golimumab and certolizumab the corresponding figures were 3.08 (0.91–10.43), 8.61 (3.55–20.86), 4.34 (3.30–5.70), 1.56 (0.93–2.60) and 5.95 (3.97–8.92), respectively (figure 2). These results suggest that infliximab and golimumab do not differ significantly from the control. In this comparison golimumab appears to be inferior in efficacy compared to etanercept, adalimumab and certolizumab even after accounting for the possible bias. TNF-blockers as a group were found to be significantly more efficacious than control at all time points using ACR 20, 50 or 70 as outcome measures. The risk ratios observed at 12 months were significantly lower than those at three or six months.

We found some evidence that the duration of RA predicts the efficacy of TNF-blocker treatment. Patients on either infliximab or adalimumab with disease duration more than 2 years were more likely to reach ACR 20, 50 and 70 at 12 months compared to controls than patients with disease duration less than two years (table 2).

**Table 2:** Meta-analysis of the efficacy of TNF-blockers compared to control(RR, 95% CI).

	ACR 20 3kk	ACR 50 3kk	ACR 70 3kk	ACR 20 6kk	ACR 50 6kk	ACR 70 6kk	ACR 20 12kk	ACR 50 12kk	ACR 70 12kk
**TNF-blocker vs. control** (including all combinations and doses of tnf-blocker vs. any control)									
Infliximab	**2.51 (1.51–4.15)** ^2^	**4.44 (1.77–11.16) ^2^**	**12.92 (1.81–92.02) ^2^**	**1.89 (1.00–3.56) ^2^**	3.08 (0.91–10.43) ^2^	5.17 (0.61–43.54) ^2^	1.70 (0.86–3.38) ^3^	**2.24 (1.11–4.50) ^3^**	**2.71 (1.09–6.70) ^3^**
Etanercept	**2.07 (1.25–3.42) ^5^**	**3.96 (1.55–10.14) ^5^**	**3.14 (1.93–5.10) ^5^**	**3.72 (1.91–7.24) ^2^**	**8.61 (3.55–20.86) ^2^**	**11.40 (2.21–58.65) ^2^**	**1.15 (1.03–1.29) ^3^**	**1.41 (1.26–1.57) ^2^**	**1.74 (1.44–2.09) ^2^**
Adalimumab	**3.40 (1.79–6.48) ^3^**	**5.42 (1.75–16.80) ^3^**	**8.25 (2.33–29.19) ^3^**	**2.53 (1.87–3.43) ^5^**	**4.34 (3.30–5.70) ^5^**	**5.44 (3.03–9.76) ^5^**	1.56 (0.62–3.90) ^2^	2.18 (0.54–8.83) ^2^	2.47 (0.61–10.02) ^2^
Golimumab	**1.56 (1.24–1.97) ^2^**	**3.03 (1.82–5.04) ^2^**	**2.91 (1.21–7.00) ^2^**	1.42 (0.95–2.12) ^2^	1.56 (0.93–2.60) ^2^	1.72 (0.74–3.99) ^2^	n/a	n/a	n/a
Certolizumab	n/a	n/a	n/a	**3.67 (1.35–9.96) ^3^**	**5.95 (3.97–8.92) ^3^**	**8.12 (3.96–16.63) ^3^**	n/a	n/a	n/a
Combined	**2.24 (1.63–3.08) ^12^**	**4.16 (2.44–7.09) ^12^**	**3.59 (2.42–5.33) ^12^**	**2.50 (1.90–3.30) ^14^**	**4.07 (2.70–6.13) ^14^**	**4.94 (2.80–8,71) ^14^**	**1.35 (1.14–1.59) ^8^**	**1.76 (1.36–2.27) ^7^**	**1.94 (1.46–2.57) ^7^**
**TNF-blocker vs. control** (early RA, disease duration ≤2 years)									
Infliximab	3.00 (0.79–11.44) ^1^	13.00 (0.83–203.83) ^1^	13.00 (0.83–203.83) ^1^	n/a	n/a	n/a	**1.20 (1.07–1.36) ^2^**	**1.52 (1.26–1.82) ^2^**	**1.68 (1.32–2.14) ^2^**
Etanercept	n/a	n/a	n/a	n/a	n/a	n/a	**1.20 (1.04–1.38) ^2^**	**1.44 (1.24–1.68) ^1^**	**1.71 (1.35–2.16) ^1^**
Adalimumab	n/a	n/a	n/a	n/a	n/a	n/a	1.01 (0.90–1.13) ^1^	1.12 (0.96–1.31) ^1^	1.28 (1.02–1.60) ^1^
Golimumab	n/a	n/a	n/a	n/a	n/a	n/a	n/a	n/a	n/a
Certolizumab	n/a	n/a	n/a	n/a	n/a	n/a	n/a	n/a	n/a
Combined	3.00 (0.79–11.44) ^1^	13.00 (0.83–203.83) ^1^	13.00 (0.83–203.83) ^1^	n/a	n/a	n/a	**1.15 (1.04–1.28) ^5^**	**1.36 (1.14–1.62) ^4^**	**1.54 (1.30–1.83) ^4^**
**TNF-blocker vs. control** (Estabilished and late RA, disease duration >2 years)									
Infliximab	**2.44 (1.41–4.20) ^1^**	**3.88 (1.46–10.31) ^1^**	12.83 (0.78–211.37) ^1^	**1.89 (1.00–3.56) ^2^**	3.08 (0.91–10.43) ^2^	5.17 (0.61–43.54) ^2^	**3.17 (2.05–4.89) ^1^**	**4.27 (2.18–8.38) ^1^**	**9.19 (2.30–36.73) ^1^**
Etanercept	**2.07 (1.25–3.42) ^5^**	**3.96 (1.55–10.14) ^5^**	**3.14 (1.93–5.10) ^5^**	**3.72 (1.91–7.24) ^2^**	**8.61 (3.55–20.86) ^2^**	**11.40 (2.21–58.65) ^2^**	1.07 (0.98–1.17) ^1^	**1.36 (1.15–1.61) ^1^**	**1.79 (1.33–2.41) ^1^**
Adalimumab	**3.40 (1.79–6.48) ^3^**	**5.42 (1.75–16.80) ^3^**	**8.25 (2.33–29.19) ^3^**	**2.53 (1.87–3.43) ^5^**	**4.34 (3.30–5.70) ^5^**	**5.44 (3.03–9.76) ^5^**	**2.46 (1.87–3.22) ^1^**	**4.37 (2.77–6.91) ^1^**	**5.15 (2.60–10.22) ^1^**
Golimumab	**1.56 (1.24–1.97) ^2^**	**3.03 (1.82–5.04) ^2^**	**2.91 (1.21–7.00) ^2^**	1.42 (0.95–2.12) ^2^	1.56 (0.93–2.60) ^2^	1.72 (0.74–3.99) ^2^	n/a	n/a	n/a
Certolizumab	n/a	n/a	n/a	**3.67 (1.35–9.96) ^3^**	**5.95 (3.97–8.92) ^3^**	**8.12 (3.96–16.63) ^3^**	n/a	n/a	n/a
Combined	**2.22 (1.60–3.07) ^11^**	**4.01 (2.34–6.87) ^11^**	**3.50 (2.35–5.21) ^11^**	**2.50 (1.90–3.30) ^14^**	**4.07 (2.70–6.13) ^14^**	**4.94 (2.80–8,71) ^14^**	2.00 (0.83–4.81) ^3^	**2.86 (1.07–7.65) ^3^**	**3.84 (1.39–10.61) ^3^**

**Bolded** risk ratios highlight statistically significant results (P<0.05), TNF = Tumour Necrosis Factor.

Superscript indicates the number of RCTs included in the comparison, RA = Rheumatoid Arthritis.

#### TNF-blocker + MTX vs. MTX

Patients on combination therapy had significantly higher ACR outcomes than ones treated with MTX alone at all time points (table 3). A statistically significant difference was revealed between ACR 20 risk ratios of certolizumab (CI 95% 5.08, 3.46–7.48) and golimumab (1.61, 0.94–2.76). However, all certolizumab studies in this comparison were potentially biased. In a subanalysis of trials with patients who had previously used MTX, the results were similar. In comparison to MTX, golimumab combination therapy was still inferior in ACR 20 efficacy at 6 months to certolizumab combination therapy, with risk ratios of 2.14 (1.59–2.89) and 5.08 (3.46–7.48), respectively. At six months patients previously naïve to MTX are statistically significantly less likely to reach either ACR 20, 50 or 70 treatment responses compared to patients who had already been previously treated with MTX. The combination of TNF-blocker and MTX was superior in efficacy to monotherapy with a TNF-blocker at almost all time points (table 4).

**Table 3:** Meta-analysis of the efficacy of combination TNF-blocker and MTX compared to MTX (RR, 95% CI).

**TNF-blocker + MTX vs. MTX** (both MTX naive patients and patients with previous experience with MTX)									
Infliximab	**2.51 (1.51–4.15) ^2^**	**4.44 (1.77–11.16) ^2^**	**12.92 (1.81–92.02) ^2^**	1.89 (1.00–3.56) ^2^	3.08 (0.91–10.43) ^2^	5.17 (0.61–43.54) ^2^	1.70 (0.86–3.38) ^3^	**2.24 (1.11–4.50) ^3^**	**2.71 (1.09–6.70) ^3^**
Etanercept	**1.77 (1.06–2.96) ^3^**	**4.40 (0.98–19.73) ^3^**	**4.24 (2.43–7.40) ^3^**	**2.67 (1.44–4.94) ^1^**	**11.69 (1.66–82.47) ^1^**	**9.82 (0.59–163.15) ^1^**	**1.20 (1.06–1.36) ^2^**	**1.51 (1.35–1.69) ^2^**	**1.93 (1.46–2.56) ^2^**
Adalimumab	1.63 (0.69–3.83) ^1^	2.06 (0.54–7.90) ^1^	3.97 (0.24–66.96) ^1^	**2.38 (1.52–3.72) ^3^**	**3.16 (1.29–7.69) ^3^**	**4.82 (2.43–9.57) ^3^**	1.67 (0.76–3.68) ^2^	2.39 (0.70–8.16) ^2^	2.78 (0.87–8.85) ^2^
Golimumab	**1.67 (1.32–2.12) ^2^**	**3.52 (2.10–5.90) ^2^**	**3.36 (1.36–8.29) ^2^**	1.61 (0.94–2.76) ^2^	1.78 (0.91–3.48) ^2^	1.98 (0.82–4.77) ^2^	n/a	n/a	n/a
Certolizumab	n/a	n/a	n/a	**5.08 (3.46–7.48) ^2^**	**6.43 (3.33–12.44) ^2^**	**7.87 (3.75–16.51) ^2^**	n/a	n/a	n/a
Combined	**1.78 (1.38–2.30) ^8^**	**3.54 (1.97–6.34) ^8^**	**4.23 (2.69–6.67) ^8^**	**2.48 (1.76–3.49) ^10^**	**3.37 (2.09–5.44) ^10^**	**4.23 (2.35–7.60) ^10^**	**1.45 (1.20–1.74) ^7^**	**1.84 (1.46–2.31) ^7^**	**2.10 (1.62–2.71) ^7^**
**TNF-blocker + MTX vs. MTX** (patients with previous experience with MTX)									
Infliximab	**2.44 (1.41–4.20) ^1^**	**3.88 (1.46–10.31) ^1^**	12.83 (0.78–211.37) ^1^	1.89 (1.00–3.56) ^2^	3.08 (0.91–10.43) ^2^	5.17 (0.61–43.54) ^2^	**3.17 (2.05–4.89) ^1^**	**4.27 (2.18–8.38) ^1^**	**9.19 (2.30–36.73) ^1^**
Etanercept	**1.77 (1.06–2.96) ^3^**	4.40 (0.98–19.73) ^3^	**4.24 (2.43–7.40) ^3^**	**2.67 (1.44–4.94) ^1^**	**11.69 (1.66–82.47) ^1^**	9.82 (0.59–163.15) ^1^	**1.13 (1.03–1.24) ^1^**	**1.60 (1.35–1.90) ^1^**	**2.27 (1.67–3.09) ^1^**
Adalimumab	1.63 (0.69–3.83) ^1^	2.06 (0.54–7.90) ^1^	3.97 (0.24–66.96) ^1^	**2.38 (1.52–3.72) ^3^**	**3.16 (1.29–7.69) ^3^**	**4.82 (2.43–9.57) ^3^**	**2.46 (1.87–3.22) ^1^**	**4.37 (2.77–6.91) ^1^**	**5.15 (2.60–10.22) ^1^**
Golimumab	**1.67 (1.32–2.12) ^2^**	**3.52 (2.10–5.90) ^2^**	**3.36 (1.36–8.29) ^2^**	**2.14 (1.59–2.89) ^1^**	**2.57 (1.60–4.14) ^1^**	**3.31 (1.50–7.28) ^1^**	n/a	n/a	n/a
Certolizumab	n/a	n/a	n/a	**5.08 (3.46–7.48) ^2^**	**6.43 (3.33–12.44) ^2^**	**7.87 (3.75–16.51) ^2^**	n/a	n/a	n/a
Combined	**1.75 (1.35–2.27) ^7^**	**3.34 (1.86–6.00) ^7^**	**4.10 (2.59–6.51) ^7^**	**2.69 (1.93–3.75) ^9^**	**3.37 (2.38–5.98) ^9^**	**4.70 (3.07–7.19) ^9^**	2.04 (0.85–4.86) ^3^	**3.01 (1.26–7.21) ^3^**	**4.05 (1.76–9.32) ^3^**
**TNF-blocker + MTX vs. MTX** (MTX naive patients exclusively)									
Infliximab	3.00 (0.79–11.44) ^1^	13.00 (0.83–203.83) ^1^	13.00 (0.83–203.83) ^1^	n/a	n/a	n/a	**1.20 (1.07–1.36) ^2^**	**1.52 (1.26–1.82) ^2^**	**1.68 (1.32–2.14) ^2^**
Etanercept	n/a	n/a	n/a	n/a	n/a	n/a	**1.28 (1.16–1.42) ^1^**	**1.44 (1.24–1.68) ^1^**	**1.71 (1.35–2.16) ^1^**
Adalimumab	n/a	n/a	n/a	n/a	n/a	n/a	**1.16 (1.03–1.31) ^1^**	**1.35 (1.15–1.59) ^1^**	**1.64 (1.29–1.92) ^1^**
Golimumab	n/a	n/a	n/a	**1.25 (1.04–1.49) ^1^**	1.31 (0.99–1.72) ^1^	1.35 (0.89–2.05) ^1^	n/a	n/a	n/a
Certolizumab	n/a	n/a	n/a	n/a	n/a	n/a	n/a	n/a	n/a
Combined	3.00 (0.79–11.44) ^1^	13.00 (0.83–203.83) ^1^	13.00 (0.83–203.83) ^1^	**1.25 (1.04–1.49) ^1^**	1.31 (0.99–1.72) ^1^	1.35 (0.89–2.05) ^1^	**1.22 (1.14–1.30) ^4^**	**1.43 (1.30–1.57) ^4^**	**1.67 (1.46–1.92) ^4^**

**Bolded** risk ratios highlight statistically significant results (P<0.05), RA = Rheumatoid Arthritis, MTX = Methotrexate.

Superscript indicates the number of RCTs included in the comparison, TNF = Tumour Necrosis Factor.

**Table 4:** Meta-analysis of the efficacy of combination TNF-blocker and MTX compared to TNF-blocker monotherapy (RR, 95% CI).

**TNF-blocker + MTX vs. TNF-blocker**									
Infliximab	n/a	n/a	n/a	n/a	n/a	n/a	n/a	n/a	n/a
Etanercept	**1.14 (1.01–1.29) ^1^**	1.27 (1.00–1.62) ^1^	**2.34 (1.46–3.77) ^1^**	n/a	n/a	n/a	**1.12 (1.02–1.23) ^1^**	**1.43 (1.22–1.69) ^1^**	**1.77 (1.34–2.33) ^1^**
Adalimumab	n/a	n/a	n/a	n/a	n/a	n/a	**1.35 (1.19–1.54) ^1^**	**1.52 (1.28–1.80) ^1^**	**1.77 (1.40–2.25) ^1^**
Golimumab	1.25 (0.99–1.58) ^1^	**1.58 (1.06–2.35) ^1^**	1.49 (0.72–3.09) ^1^	1.40 (1.00–1.96) ^2^	1.41 (0.94–2.11) ^2^	**1.53 (1.08–2.17) ^2^**	n/a	n/a	n/a
Certolizumab	n/a	n/a	n/a	n/a	n/a	n/a	n/a	n/a	n/a
Combined	**1.16 (1.05–1.29) ^2^**	**1.35 (1.09–1.66) ^2^**	**2.04 (1.36–3.07) ^2^**	1.40 (1.00–1.96) ^2^	1.41 (0.94–2.11) ^2^	**1.53 (1.08–2.17) ^2^**	**1.22 (1.01–1.49) ^1^**	**1.47 (1.31–1.66) ^1^**	**1.77 (1.48–2.12) ^1^**

**Bolded** risk ratios highlight statistically significant results (P<0.05), RA = Rheumatoid Arthritis, MTX = Methotrexate.

Superscript indicates the number of RCTs included in the comparison, TNF = Tumour Necrosis Factor.

#### TNF-blocker monotherapy vs. MTX

There are no trials comparing monotherapy of infliximab to MTX, but combined results with the remaining four other TNF-blockers show that while the risk ratios favour the TNF-blocker, the results do not reach statistical significance at any time point using ACR 20, 50 or 70 as outcome measures. Stratifying RTCs by previous exposure to MTX does not show any statistically significant differences in the treatment response to TNF-blocker monotherapy between these two groups.

#### TNF-blocker monotherapy vs. placebo

All four TNF-blockers were more efficacious than placebo with the estimates of risk ratios ranging from 2.74 (CI 95% 1.76–4.26) – 12.31 (1.64–92.41). There were no statistically significant differences in efficacy between individual substances in this comparison or, alternatively, the meta-analysis was underpowered to reveal them.

#### High doses of TNF-blockers vs. normal doses

The final meta-analysis compared higher than regular doses of TNF-blockers to normal doses (table 5). Both patients using high and normal doses had to be on concomitant MTX or *on* TNF-blocker monotherapy. Increasing the dose of TNF-blocker provided no additional efficacy compared to regular doses except 12 months with possibly biased results excluded.

**Table 5:** Meta-analysis of the efficacy of high doses of TNF-blockers compared to normal doses of TNF-blockers.

**High doses of TNF-blocker vs. normal doses** (both high and normal dose of TNF-blocker in combination with MTX or vice versa)									
Infliximab	0.86 (0.61–1.22) ^1^	1.15 (0.66–2.02) ^1^	0.96 (0.30–3.11) ^1^	1.07 (0.84–1.36) ^1^	1.09 (0.72–1.63) ^1^	1.57 (0.72–3.40) ^1^	1.16 (0.93–1.46) ^2^	1.36 (0.84–2.19) ^2^	1.43 (0.81–2.52) ^2^
Etanercept	n/a	n/a	n/a	n/a	n/a	n/a	n/a	n/a	n/a
Adalimumab	1.26 (0.93–1.71) ^1^	1.27 (0.74–2.16) ^1^	0.70 (0.33–1.47) ^1^	1.08 (0.92–1.26) ^3^	1.12 (0.71–1.76) ^3^	1.08 (0.68–1.70) ^3^	n/a	n/a	n/a
Golimumab	1.02 (0.84–1.25) ^2^	0.81 (0.58–1.13) ^2^	0.93 (0.40–2.15) ^2^	1.00 (0.87–1.15) ^2^	0.90 (0.71–1.13) ^2^	0.75 (0.52–1.07) ^2^	n/a	n/a	n/a
Certolizumab	n/a	n/a	n/a	1.02 (0.93–1.12) ^2^	1.06 (0.91–1.22) ^2^	0.84 (0.60–1.19) ^2^	n/a	n/a	n/a
Combined	1.04 (0.90–1.21) ^4^	0.96 (0.75–1.24) ^4^	0.82 (0.52–1.31) ^4^	1.03 (0.97–1.10) ^8^	1.02 (0.90–1.15) ^8^	0.91 (0.74–1.10) ^8^	1.16 (0.93–1.46) ^2^	1.36 (0.84–2.19) ^2^	1.43 (0.81–2.52) ^2^

**Bolded** risk ratios highlight statistically significant results (P<0.05), RA = Rheumatoid Arthritis, MTX = Methotrexate.

Superscript indicates the number of RCTs included in the comparison, TNF = Tumour Necrosis Factor.

### Sensitivity analyses

The sensitivity analyses based on the results of the bias assessments did not reveal any statistically significant bias on the efficacy results. Occasionally, however, the statistical significance between intervention and control groups disappeared due to reduced number of studies. In the sensitivity analyses, the estimate of the risk ratio decreased, increased or remained the same in 52%, 45% and 3% of cases, respectively. In some cases there were no clearly unbiased RCTs in a comparison, thus making it impossible to perform the sensitivity analysis. Significant heterogeneity was present in the first analysis comparing any intervention to any control. Heterogeneity diminished as the comparisons were stratified into smaller comparisons.

To investigate the possible effect of study patients' baseline disease activity on efficacy, two additional analyses were performed. Using ACR 50 at six months as outcome and stratifying trials into two categories by the number of swollen joints or Health Assessment Questionnaire (HAQ) score revealed no statistical differences between the subgroups. Trials with low swollen joint count and low HAQ score had risk ratios of 3.43 (CI 95% 2.03–5.78) and 3.68 (2.11–6.42), respectively, whereas trials with high swollen joint count and high HAQ score had risk ratios of 5.15 (2.72–9.75) and 4.64 (2.59–8.31), respectively.

### Safety

#### TNF-blocker vs. control

**Figure 3:**
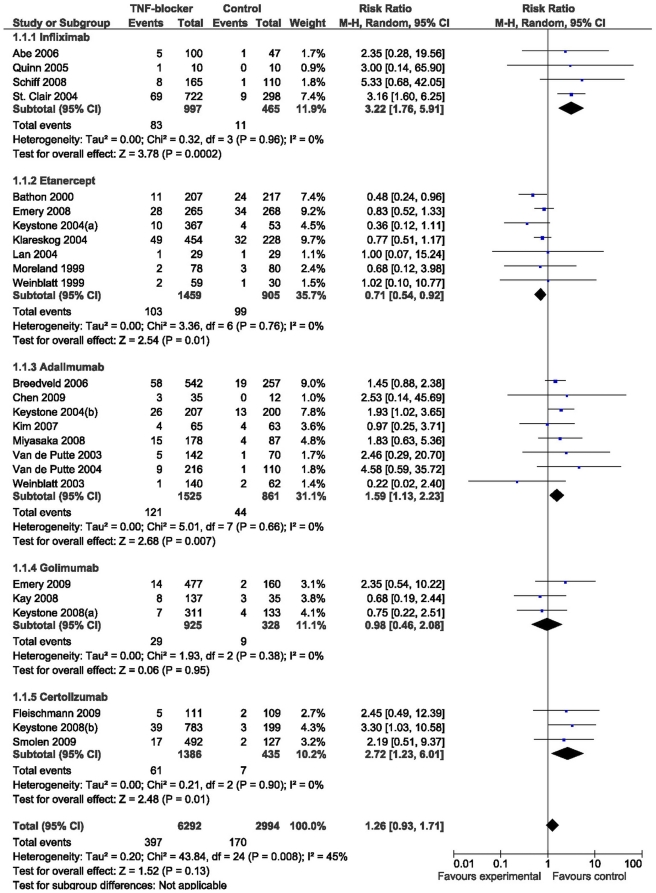
Forest plot of the number of discontinuations due to an adverse event.

The primary safety endpoint of the systematic review was the discontinuation of study due to adverse events. There were 25 studies with 6292 patients in the intervention and 2994 in the control group in this analysis (table 6). As a group, the TNF-blockers did not statistically significantly differ from the control (RR 1.26, CI 95% 0.93–1.71). While the patients on infliximab (3.22, 1.76–5.91), adalimumab (1.59, 1.13–2.23), and certolizumab (2.72, 1.23–6.01), had an increased risk to discontinue, the patients on etanercept (0.71, 0.54–0.92) had a decreased risk (figure 3). Patients using certolizumab had a higher risk to experience a serious adverse event than patients on etanercept with risk ratios of 2.24 (1.38–3.63) and 0.90 (0.68–1.20), respectively. Infliximab, etanercept and golimumab increased the likelihood of an injection or infusion reaction while adalimumab and certolizumab did not statistically significantly differ from the controls in this respect although their risk ratios leaned to the same direction.

**Table 6:** Meta-analysis of the safety of TNF-blockers in different comparisons (RR, 95% CI).

	Discontinuation due to adverse event	All adverse events	Serious adverse events	All infections	Serious infections	Injection or infusion reactions
**TNF-blocker vs. control**						
Infliximab	**3.22 (1.76–5.91)^4^**	1.02 (0.93–1.13)^2^	1.01 (0.70–1.47)^4^	1.51 (0.92–2.47)^3^	1.45 (0.63–3.35)^3^	**1.76 (1.03–3.03)^3^**
Etanercept	**0.71 (0.54–0.92)^7^**	1.02 (0.97–1.07)^1^	0.90 (0.68–1.20)^3^	0.88 (0.65–1.20)^2^	0.87 (0.48–1.58)^3^	**4.46 (3.13–6.36)^5^**
Adalimumab	**1.59 (1.13–2.23)^8^**	1.04 (0.94– 1.15)^4^	1.03 (0.71–1.49)^5^	1.34 (0.93–1.94)^4^	2.89 (0.68–12.36)^4^	3.08 (0.94–10.13)^7^
Golimumab	0.98 (0.46– 2.08)^3^	1.05 (0.97– 1.13)^3^	1.24 (0.57–2.73)^3^	1.03 (0.74–1.44)^3^	1.41 (0.53–3.72)^3^	**2.20 (1.15–4.19)^3^**
Certolizumab	**2.72 (1.23–6.01)^3^**	1.15 (0.89– 1.48)^2^	**2.24 (1.38–3.63)^3^**	0.62 (0.37–1.22)^1^	6.11 (0.78–47.93)^2^	1.53 (0.15–15.28)^3^
Combined	1.26 (0.93–1.71)^24^	1.04 (1.00–1.09)^14^	1.10 (0.91–1.34)^18^	1.10 (0.89–1.36)^13^	1.40 (0.93–2.10)^15^	**2.46 (1.63–3.70)^21^**
**TNF-blocker + MTX vs. MTX**						
Infliximab	**2.06 (1.05–4.07)^5^**	1.02 (0.93–1.13)^2^	1.04 (0.65–1.66)^4^	1.23 (0.94–1.61)^3^	1.45 (0.63–3.35)^3^	**1.76 (1.03–3.03)^3^**
Etanercept	0.78 (0.56–1.09)^4^	0.99 (0.94–1.03)^2^	0.85 (0.62–1.16)^3^	0.97 (0.78–1.20)^2^	0.71 (0.37–1.36)^3^	**4.44 (1.81–10.86)^4^**
Adalimumab	**1.58 (1.08**–**2.33)^5^**	1.02 (0.99–1.06)^3^	1.23 (0.49–3.10)^2^	1.20 (0.85–1.71)^3^	2.37 (0.38–14.91)^3^	1.04 (0.51–2.11)^4^
Golimumab	1.20 (0.48–3.00)^3^	1.08 (1.00–1.18)^3^	1.45 (0.66–3.16)^3^	0.99 (0.79–1.24)^3^	1.59 (0.60–4.26)^3^	**2.15 (1.11–4.15)^3^**
Certolizumab	**2.82 (1.14–6.98)^2^**	1.01 (0.84–1.22)^1^	**2.18 (1.30–3.67)^2^**	1.19 (0.82–1.73)^1^	7.38 (0.44–122.91)^1^	5.31 (0.72–39.41)^2^
Combined	**1.37 (1.01–1.87)^19^**	1.02 (0.99–1.04)^11^	1.14 (0.89–1.47)^14^	1.08 (0.97–1.20)^12^	1.28 (0.81–2.04)^13^	**2.08 (1.40–3.10)^16^**
**TNF-blocker + MTX vs. TNF-blocker**						
Infliximab	n/a	n/a	n/a	n/a	n/a	n/a
Etanercept	0.93 (0.55–1.57)^1^	0.94 (0.87–1.02)^1^	0.80 (0.51–1.26)^1^	1.13 (0.98–1.31)^1^	0.97 (0.41–11.21)^1^	**0.48 (0.30–0.77)^1^**
Adalimumab	1.26 (0.77–2.05)^2^	1.02 (0.99.1.05)^1^	n/a	n/a	3.07 (0.84–11.21)^1^	n/a
Golimumab	2.45 (0.46–12.93)^1^	**1.14 (1.03–1.26)^2^**	1.97 (0.98–3.95)^2^	0.94 (0.76–1.16)^2^	3.02 (0.88–10.30)^2^	0.83 (0.37–1.87)^2^
Certolizumab	n/a	n/a	n/a	n/a	n/a	n/a
Combined	1.19 (0.78–1.80)^4^	1.04 (0.96–1.13)^4^	1.29 (0.65–2.58)^3^	1.06 (0.93–1.21)^3^	1.83 (0.88–3.81)^4^	0.62 (0.38–1.00)^3^
**TNF-blocker vs. MTX**						
Infliximab	n/a	n/a	n/a	n/a	n/a	n/a
Etanercept	0.66 (0.40–1.07)^2^	1.06 (0.98–1.15)^1^	0.97 (0.63–1.48)^1^	0.91 (0.79–1.05)^1^	1.02 (0.43–2.41)^1^	**6.89 (3.05–8.35)^2^**
Adalimumab	1.28 (0.73–2.26)^1^	1.00 (0.97–1.04)^1^	n/a	n/a	0.40 (0.11–1.54)^1^	n/a
Golimumab	0.67 (0.19–2.36)^2^	0.97 (0.86–1.08)^2^	0.80 (0.23–2.79)^2^	1.13 (0.89–1.44)^2^	0.75 (0.17–3.36)^2^	2.94 (0.70–12.30)^2^
Certolizumab	n/a	n/a	n/a	n/a	n/a	n/a
Combined	0.81 (0.56–1.18)^5^	1.01 (0.98–1.04)^4^	0.89 (0.53–1.47)^3^	1.00 (0.84–1.19)^3^	0.78 (0.40–1.49)^4^	**5.20 (2.62–10.31)^4^**
**TNF-blocker vs. placebo**						
Infliximab	n/a	n/a	n/a	n/a	n/a	n/a
Etanercept	0.68 (0.12–3.98)^1^	n/a	n/a	n/a	n/a	**3.90 (2.09–7.27)^1^**
Adalimumab	2.27 (0.95–5.40)^3^	1.06 (0.98–1.14)^1^	0.99 (0.66–1.50)^3^	1.19 (0.86–1.64)^1^	4.15 (0.78–22.18)^2^	**7.71 (3.39–17.54)^3^**
Golimumab	n/a	n/a	n/a	n/a	n/a	n/a
Certolizumab	2.45 (0.49–12.39)^1^	**1.31 (1.08–1.59)^1^**	2.62 (0.71–9.61)^1^	n/a	4.91 (0.24–101.13)^1^	**0.33 (0.12–0.87)^1^**
Combined	1.90 (0.94–3.84)^5^	1.16 (0.88–1.53)^2^	1.13 (0.71–1.80)^4^	1.19 (0.86–1.64)^1^	4.32 (1.00–18.70)^3^	**3.69 (1.03–13.23)^5^**
**High doses of TNF-blocker vs. normal doses**						
Infliximab	1.14 (0.76–1.73)^3^	0.99 (0.78–1.25)^1^	1.19 (0.65–2.17)^3^	0.92 (0.53–1.59)^2^	1.49 (0.33–6.67)^2^	**0.73 (0.56–0.94)^2^**
Etanercept	n/a	n/a	n/a	n/a	n/a	n/a
Adalimumab	0.43 (0.17–1.05)^3^	0.94 (0.89–1.00)^1^	0.87 (0.45–1.71)^3^	0.94 (0.68–1.32)^1^	0.52 (0.13–2.03)^1^	1.31 (0.79–2.17)^4^
Golimumab	1.19 (0.54–2.61)^3^	0.92 (0.82–1.02)^3^	1.04 (0.58–1.85)^3^	0.90 (0.71–1.14)^3^	2.28 (0.83–6.27)^3^	1.52 (0.86–2.69)^3^
Certolizumab	0.87 (0.34–2.20)^2^	0.91 (0.77–1.07)^1^	1.06 (0.76–1.46)^2^	0.77 (0.57–1.06)^1^	0.76 (0.27–2.15)^1^	0.73 (0.15–3.52)^2^
Combined	0.98 (0.72–1.35)^11^	**0.93 (0.89–0.97)^6^**	1.01 (0.83–1.23)^11^	0.92 (0.79–1.07)^7^	1.10 (0.66–1.87)^7^	1.05 (0.78–1.40)^11^

**Bolded** risk ratios highlight statistically significant results (P<0.05), RA = Rheumatoid Arthritis, MTX = Methotrexate.

Superscript indicates the number of RCTs included in the comparison, TNF = Tumour Necrosis Factor.

#### TNF-blocker + MTX vs. MTX

In this comparison, etanercept no longer significantly decreased the likelihood of discontinuation due to adverse event (RR 0.78, CI 95% 0.56–1.09). Combined results from all substances now reached statistical significance (1.37, 1.01–1.87). In an analysis comparing the combination treatment to monotherapy there were only few differences between treatment groups. There was a trend of elevated risk ratios of multiple safety endpoints excluding injection reactions but these findings did not reach statistical significance. Golimumab increased the odds for an unspecified adverse event (1.14, 1.03–1.26) while others did not.

#### TNF-blocker monotherapy vs. MTX

TNF-blocker and MTX were comparable in all respects other than injection and infusion reactions (RR 5.20, CI 95% 2.62–10.31).

#### TNF-blocker monotherapy vs. placebo

The comparison of TNF-blockers and placebo showed a trend of increased risk of adverse events from TNF-blockers, but only the increase in the frequency of injection reactions was statistically significant (RR 3.69, CI 95% 1.03–13.23). In addition, certolizumab seemed to increase the risk to experience an adverse event compared to placebo (1.31, 1.08–1.26).

#### High doses of TNF-blockers vs. normal doses

Increased dose of the TNF-blockers did not increase the frequency of discontinuations due to adverse events (RR 0.98, CI 95% 0.72–1.35), but the likelihood to experience an unspecified adverse event was reduced compared to normal doses (0.93, 0.89–0.97). Patients on high doses of infliximab were less likely to suffer from infusion reactions compared to those on regular doses (0.73, 0.56–0.94).

## Discussion

### Findings of this review

Our systematic literature search identified 40 publications reporting 26 randomized controlled trials studying the efficacy and safety of TNF-blockers. The included trials were published 1999–2010. Five trials published used infliximab [49]–[59], seven etanercept [60]–[72], eight adalimumab [73]–[82], three golimumab [83]–[85] and three certolizumab [86]–[88] for the intervention. Overall, there were 6780 patients in the intervention and 3082 in the control group. The patients' characteristics varied across the included trials with mean time since diagnosis ranging from 0.5 to 13 years, HAQ score from 1.25 to 1.88 and the number of swollen and tender joints from 11 and 14.03 to 24 and 37.2, respectively.

The results of the primary efficacy endpoint suggest that infliximab and golimumab do not statistically significantly differ from control regarding efficacy in a comparison between any combination of TNF-blocker and any control. Even though the different settings and heterogeneity among the studies could have accounted for the result, the finding still raises questions. Golimumab appears to be inferior in efficacy to etanercept, adalimumab and certolizumab even after accounting for the eventual bias. Patients in golimumab trials have lower count of swollen and tender joints as well as lower HAQ score, which may explain the results to some extent, although an ad hoc analysis on the effect of patient characteristics on efficacy showed no statistical significance. Disease duration seems to predict treatment response to adalimumab and infliximab at 12 months.

In the second and third meta-analysis the efficacy of MTX and TNF-blocker combination was found to be superior to either MTX or TNF-blocker alone, respectively. The increase in the number of discontinuations due to adverse events (RR 1.37 95% 1.01–1.87) compared to MTX alone is likely to be acceptable. Patients with previous exposure to MTX were more likely to benefit from the combination therapy compared to MTX naïve patients. Compared to monotherapy with a TNF-blocker the safety of the combination treatment was equal or even improved regarding some aspects.

The fourth meta-analysis found no statistical difference between MTX and TNF-blocker monotherapy and the fifth one confirmed that TNF-blocker monotherapy was more efficacious than placebo. The last secondary efficacy meta-analysis found little benefit from increasing the dose of TNF-blockers.

In the first safety comparison between TNF-blockers and control the risk ratios reached statistical significance only in the number of patients experiencing injection or infusion reactions. Interestingly, infliximab, adalimumab and certolizumab increased the risk of discontinuation of treatment due to adverse events, but etanercept made it less likely. Certolizumab was the only TNF-blocker which increased the likelihood of experiencing a serious adverse event. While TNF-blockers as a group increased the odds to experience an injection or infusion reaction this may not be the case with adalimumab and certolizumab.

### Strengths and limitations

It could be asked, whether TNF-blocker naive and switchers should be included in the same review, because these patients could be very different. However, fifteen trials included in this systematic review stated previous TNF-blocker use as an exclusion criterion. In eight more trials it was unclear if switchers were included and only two certolizumab trials included switchers but excluded those who had had insufficient response to previous TNF-blocker treatment. However, the percentage of previous TNF-blocker users in these two trials was small (2–4%) and a sensitivity analysis was performed.

While broader comparisons with larger number of trials may be more likely to reach statistically significant results (1.00 not included in the confidence interval), their validity may be questioned. Heterogeneity introduced by combining the results of trials with different settings causes random effects model to calculate wider confidence intervals than fixed effects model would do. While reducing the possibility of type I error, it may introduce a type II error. Hence, the efficacies of TNF-blockers were compared with different controls, combinations and dosages in smaller, but more homogenous comparisons.

Results of the sensitivity analyses revealed that the source of bias in the RCTs is as likely to lead to underestimation as overestimation of the risk difference between intervention and control groups. However, the homogeneity of study population, intervention, control, outcomes and study settings are likely to be more crucial to the validity of the meta-analysis. Length of exposure was not taken into account in the safety analyses, only the difference in risk ratios between intervention and control group.

The methods used in the study were derived from the Handbook for systematic reviews of interventions by Cochrane Collaboration. The team involved in study design and execution included clinicians, methodology experts and pharmacists. Two researchers independently worked at each step and afterwards combined their results to improve the validity of the study. The meta-analyses were done using Review Manager 5.0 –software. The report was written in accordance to the PRISMA-statement (table S3).

Our systematic review and meta-analysis has some limitations. The authors of the included trials were not contacted to retrieve unpublished data. Many studies that lasted for one year or more only reported results at 12 months. The meta-analyses would have been more powered if the efficacy results had been reported at all time points. Selective reporting was included in the evaluation for bias, but we were unable to identify any bias here.

### Findings in comparison to other systematic reviews

Another systematic review and meta-analysis pooled efficacy results from different time points and found slightly different estimates for the efficacy of TNF-blockers, which is likely due to differences in study designs [12]. Several large clinical trials have been published since the aforementioned review along with the introduction of two novel TNF-blockers, certolizumab and golimumab. Our study distinguishes itself from previous systematic reviews by including larger number of clinical trials and by presenting efficacy results separately at three, six and twelve months. Our results reveal that the new substances do not offer improved efficacy or safety profile over the already existing ones. A recent systematic review concluded that certolizumab is at least as efficacious compared to older TNF-blockers [14]. However, the study did not include studies with MTX comparison nor evaluate the safety of biologic treatments. Certolizumab may be more efficacious than golimumab but may also be associated with a greater risk of serious adverse events.

A previous systematic review reached the same conclusion as we did, regarding the increased dose of TNF-blockers. Contradicting our results, they found high doses leading to two-fold risk of serious infections. In contrast to our direct approach, they however separately compared recommended and high doses of TNF-blockers to placebo [10]. Another systematic review concluded that infliximab might require an increased dosage level to reach similar efficacy as etanercept and adalimumab have [13].

### Implications for practise and research

The novel TNF-blockers may offer an alternative to older substances but do not make them obsolete. On the contrary, etanercept may be the best choice when taking into account safety profiles of the TNF-blockers. Infliximab, etanercept and adalimumab have been in clinical use for years with extensive amount of post-marketing data available. More post-marketing information is needed on certolizumab and golimumab for comprehensive pharmacovigilance.

The annual medication costs of TNF-blockers are more than 10 000€ while the MTX treatment costs less than 100€ per year. Subgroup analysis in table 3 suggests that considering the high expenses of biologics, the treatment of RA could be initiated with MTX while combining TNF-blockers to ongoing treatment in patients with insufficient response to MTX. Even though safety was not compromised, it might not be cost-effective to use high doses of TNF-blockers. Given the limited resources in healthcare systems our results may help clinicians and decision makers to get most out of the expensive, but efficacious treatment.

The next step could be to analyze the efficacy and safety of not just TNF-blockers, but all biologics in a large systematic review and meta-analysis. One randomized clinical trial included in our systematic review actually compared abatacept to infliximab [55]. However, a systematic review is indicated to summarise the evidence.

## Supporting Information

Table S1 **Search strategy to (Ovid^®^) Medline.**
(DOCX)

Table S2 **Description of studies included in the systematic review and meta-analysis.** 1 = Evaluation based on 28 joints. 2 = Baseline data. 3 = Evaluation based on 71 joints. 4 = Values in median. 5 = placebo switched to active medication at 6 months. Ada = Adalimumab. Cer = Certolizumab pegol. Eta = Etanercept. Gol = Golimumab. Inf = Iinfliximab. MTX = methotrexate.
(DOCX)

Table S3 **PRISMA Checklist of items to include when reporting a systematic review or meta-analysis.**
(DOCX)
