# Supplementary material for: Systematic Review and Meta-Analysis of the Efficacy and Safety of Existing TNF Blocking Agents in Treatment of Rheumatoid Arthritis
Source: PLoS One. 2012 Jan 17;7(1):e30275. doi: 10.1371/journal.pone.0030275 (PMC3260264; doi:10.1371/journal.pone.0030275)
Supplement: Table S1 — Search strategy to (Ovid®) Medline. (DOCX) [file pone.0030275.s001.docx]

| Ovid MEDLINE(R), Ovid MEDLINE(R) In-Process (5.2.2010) | | |
| --- | --- | --- |
| # ID | Search term | Search results |
| 1 | (rheumatoid adj1 arthritis).mp. | 85757 |
| 2 | tnf*.mp. | 91740 |
| 3 | tumo?r necrosis factor*.mp. | 105378 |
| 4 | antitnf*.mp. | 22 |
| 5 | anti-tnf*.mp. | 4655 |
| 6 | antitumo?r necrosis factor*.mp. | 233 |
| 7 | anti-tumo?r necrosis factor*.mp. | 1724 |
| 8 | (infliximab* or remicade* or cA2*).mp. | 117680 |
| 9 | (etanercept* or enbrel* or p75TNFR-Fc*).mp | 2430 |
| 10 | (adalimumab* or humira* or D2E7*).mp. | 1456 |
| 11 | (certolizumab* or cimzia* or CDP870*).mp. | 173 |
| 12 | (golimumab* or simponi* or CNTO-148*).mp. | 56 |
| 13 | 2 or 3 or 4 or 5 or 6 or 7 or 8 or 9 or 10 or 11 or 12 | 238383 |
| 14 | random*.mp. | 630336 |
| 15 | rct*.mp. | 8095 |
| 16 | ((single* or double* or trebl* or tripl*) adj1 (blind* or mask*)).mp. | 141997 |
| 17 | placebo*.mp. | 135062 |
| 18 | (clinical adj trial*).mp. | 644131 |
| 19 | (meta adj1 analy*).mp. | 41768 |
| 20 | metaanaly*.mp. | 1060 |
| 21 | (systematic* adj3 (review* or overview* or litera* or search*)).mp | 27972 |
| 22 | 14 or 15 or 16 or 17 or 18 or 19 or 20 or 21 | 1065142 |
| 23 | 1 and 13 and 22 | 1556 |
