# Supplementary material for: Systematic Review and Meta-Analysis of the Efficacy and Safety of Existing TNF Blocking Agents in Treatment of Rheumatoid Arthritis
Source: PLoS One. 2012 Jan 17;7(1):e30275. doi: 10.1371/journal.pone.0030275 (PMC3260264; doi:10.1371/journal.pone.0030275)
Supplement: Table S2 — Description of studies included in the systematic review and meta-analysis. 1 = Evaluation based on 28 joints. 2 = Baseline data. 3 = Evaluation based on 71 joints. 4 = Values in median. 5 = placebo switched to active medication at 6 months. Ada = Adalimumab. Cer = Certolizumab pegol. Eta = Etanercept. Gol = Golimumab. Inf = Iinfliximab. MTX = methotrexate. (DOCX) [file pone.0030275.s002.docx]

| Study and additional publications | Intervention (I) and control groups (C) | No of pa-tients | Disease duration (years) | No of swollen joints | No of tender joints | HAQ | Previous MTX use | MTX dose (mg/vk) | Primary clinical outcome | RCT duration |
| --- | --- | --- | --- | --- | --- | --- | --- | --- | --- | --- |
| **Abe 2006^49^** | I_1_: 3mg/kg Inf + MTX | 49 | 9,1 | 15,1 | 19 | n/a | Yes | 7,1^2^ | ACR 20 14wk | 14 weeks |
|  | I_2_: 10mg/kg Inf+ MTX | 51 | 7,1 | 13,2 | 18,7 | n/a | Yes | 7,1^2^ |  |  |
|  | C_1_: MTX + placebo | 47 | 7,5 | 13,5 | 17,8 | n/a | Yes | 7,4^2^ |  |  |
|  | *Inf at weeks 0, 2 and 6* |  |  |  |  |  |  |  |  |  |
| **Maini 1999^53^** | I_1_: Inf 3mg/kg+MTX e8w | 86 | 10 | 22 | 32 | 1.8 | Yes | 16 | ACR 20 30wk | 30 weeks |
| Lipsky 2000^51^ | I_2_: Inf 3mg/kg+MTX e4w | 86 | 9 | 21 | 31 | 1.7 | Yes | 16 |  |  |
| Maini 2004^52^ | I_3_: Inf 10mg/kg+MTX e8w | 87 | 11 | 23 | 32 | 1.7 | Yes | 16 |  |  |
| Smolen 2005^56^ | I_4_: Inf 10mg/kg+MTX e4w | 81 | 12 | 24 | 34 | 1.7 | Yes | 17 |  |  |
|  | C_1_: Placebo + MTX | 88 | 11 | 21 | 31 | 1.7 | Yes | 16 |  |  |
| **Quinn 2005^54^** | I_1_: Inf 3mg/kg+MTX | 10 | 0,62 | n/a | n/a | 1,3 | No | 15 | None | 12 months |
|  | C_1_: Placebo + MTX | 10 | 0,62 |  |  |  |  |  |  |  |
|  | *Inf at weeks 0, 2 and 6, then every 8 weeks* |  |  |  |  |  |  |  |  |  |
| **Schiff 2008^55^** | I_1_: Inf 3mg/kg + MTX | 165 | 7,3 | 20,3 | 31,7 | 1,7 | Yes | 16,3 | DAS 28 6kk (abatacept vs.infliximab) | 12 months^5^ |
| Bessette 2007^50^ | C_1_: Placebo + MTX | 110 | 4 | , | 8 | 20,1 | 30,3 | 1,8 |  |  |
| Van Vollen-hoven 2008^59^ | *Inf at weeks 0, 2 and 6, then every 8 weeks* |  |  |  |  |  |  |  |  |  |
| **St. Clair 2004^58^** | I_1_: Inf 3mg/kg+ MTX | 359 | 0,8 | 21 | 32 | No | 1,5 | 15,5 | 54 weeks | ACR-N 54 wk |
| Smolen 2006^57^ | I_2_: Inf 10mg/kg + MTX | 363 | 0,9 | 22 | 33 | 1,5 | No | 14,9 |  |  |
|  | C_1_: Placebo + MTX | 282 | 0,9 | 22 | 34 | 1,5 | No | 15,1 |  |  |
|  | *Inf at weeks 0, 2 and 6, then every 8 weeks* |  |  |  |  |  |  |  |  |  |
| **Bathon 2000^61^** | I_1_: 25mg Eta | 207 | 1 | 24 | 31 | n/a | No |  | ACR-N 0-6mo | 12 months |
| Bathon 2003^60^ | C_1_: MTX + placebo | 217 | 1 | 24 | 30 | n/a | No | 19 |  |  |
| Genovese 2002^64^ | *Eta twice a week* |  |  |  |  |  |  |  |  |  |
| **Emery 2008^62^** | I_1_: Eta 50mg + | 274 | 0,733 | 17,1 | 25,1 | 1,7 | No | 16,8 | DAS remission 52wk | 52 weeks |
| Emery 2010^63^ | C_1_: MTX + placebo | 268 | 0,775 | 17,6 | 24,8 | 1,6 | No | 19.6 (wk 8) |  |  |
|  | *Eta once a week* |  |  |  |  |  |  |  |  |  |
| **Keystone 2004^66^** | I_1_: Eta 50mg | 214 | 9 | 19.2 | 26^3^ | 1,4 | No/yes | 14.3(53%) | ACR 20 8 wk | 16 weeks |
|  | I_2_: Eta 25mg | 153 | 8,2 | 19.2 | 29.2^3^ | 1,4 | No/yes | 15.0(52%) |  |  |
|  | C_1_: Placebo  Some of the patients on MTX | 53 | 10,8 | 19.2 | 24.6^3^ | 1,4 | No/yes | 13.8(55%) |  |  |
| **Klareskog 2004^67^** | I_1_: Eta 25mg | 223 | 6,3 | 23,0 | 35 | 1.7 | No/yes | 16,9 | ACR-N wk 24 | 52 weeks |
| Kavanaugh 2008^65^ | I_2_: Eta 25mg + MTX | 231 | 6,8 | 22,1 | 34,2 | 1.8 | No/yes | 17,2 |  |  |
| Van der Heijde 2006^71^ | C_1_: Placebo + MTX | 228 | 6,8 | 22,6 | 33,1 | 1.7 | No/yes | (wk 8) |  |  |
| Heijde 2007^70^ | *Eta twice a week* |  |  |  |  |  |  |  |  |  |
| **Lan 2004^68^** | I_1_: Eta 25mg + MTX | 29 | n/a | 13,21^1^ | 14,03^1^ | 0,99 | Yes | 12,5-20 | SJC and TJC | 12 weeks |
|  | C_1_: Placebo + MTX | 29 | n/a | 14.45^1^ | 16.00^1^ | 1,23 | Yes | 12,5-20 |  |  |
|  | *Eta twice a week* |  |  |  |  |  |  |  |  |  |
| **Moreland 1999^69^** | I_1_: Eta 25mg | 78 | 11 | 25 | 33 | 1,6 | No/yes |  | ACR 20 and 50 3 and 6mo | 6 months |
|  | C_1_: Placebo | 80 | 12 | 25 | 35 | 1,7 | No/yes |  |  |  |
|  | *Eta twice 2 week* |  |  |  |  |  |  |  |  |  |
| **Weinblatt 1999^72^** | I_1_: Eta 25mg + MTX | 59 | 13 | 20^4^ | 28^4^ | 1,5^4^ | Yes | 19 | Endpoints not specified | 24 weeks |
|  | C_1_: Placebo + MTX | 30 | 13 | 17^4^ | 28^4^ | 1,5^4^ | Yes | 18 |  |  |
|  | *Eta twice a week* |  |  |  |  |  |  |  |  |  |
| **Breedveld 2006^73^** | I_1_: Ada 40 mg + MTX | 274 | 0,7 | 21,8 | 31,8 | 1,6 | No | 0 | ACR 50 12mo | 2 years |
|  | I_2_: Ada 40 mg + placebo | 268 | 0,7 | 21,1 | 30,7 | 1,5 | No | 16.3 |  |  |
|  | C_1_: MTX + placebo | 257 | 0,8 | 22,1 | 32,3 | 1,5 | No | 16.9 |  |  |
|  | *Ada every other week* |  |  |  |  |  |  |  |  |  |
| **Chen 2009^74^** | I_1_: Ada 40mg + MTX | 35 | 6,2 | 21,9 | 32,5 | 1,7 | Yes | 10-15 | ACR 20 12wk | 12 weeks |
|  | C_1_: MTX + placebo | 12 | 8,3 | 24,1 | 37,2 | 1,8 | Yes | 10-15 |  |  |
|  | *Ada every other week* |  |  |  |  |  |  |  |  |  |
| **Keystone 2004^77^** | I_1_: Ada 40mg + MTX | 207 | 11 | 19,3 | 27,3 | 1,45 | Yes | 16.7 | ACR 20 wk 24 | 52 weeks |
| Jamal 2009^75^ | C_1_: Placebo + MTX | 200 | 10,9 | 19,0 | 28,1 | 1,48 | Yes | 16.7 | HAQ wk 54 |  |
| Keystone 2003^76^ | *Ada every other week* |  |  |  |  |  |  |  |  |  |
| **Kim 2007^78^** | I_1_: Ada 40mg + MTX | 65 | 6,8 | 12,2 | 19,2 | 1,4 | Yes | 16,6^2^ | ACR 20 wk 24 | 24 weeks |
|  | C_1_: Placebo + MTX | 63 | 6,9 | 12,8 | 20,3 | 1,3 | Yes | 16,3^2^ |  |  |
| **Miyasaka 2008^79^** | I_1_: Ada 40mg | 91 | 9,9 | 19,1 | 24,4 | 1,64 | No/yes |  | ACR 20 wk24 | 24 weeks |
|  | I_2_: Ada 80mg | 87 | 9,5 | 20,8 | 24,9 | 1,77 | No/yes |  |  |  |
|  | C_1_: Placebo | 87 | 8,4 | 19,3 | 23,7 | 1,39 | No/yes |  |  |  |
|  | *Ada every other week* |  |  |  |  |  |  |  |  |  |
| **Van de Putte 2003^80^** | I_1_: Ada 40mg | 70 | 10 | 18,7 | 31,0 | 1,74 | No/yes |  | ACR 20 12wk | 12 weeks |
|  | I_2_: Ada 80mg | 72 | 10,1 | 19,6 | 32,5 | 1,66 | No/yes |  |  |  |
|  | C_1_: Placebo | 70 | 9,4 | 19,8 | 30,9 | 1,63 | No/yes |  |  |  |
|  | *Ada every other week* |  |  |  |  |  |  |  |  |  |
| **Van de Putte 2004^81^** | I_1_: Ada 40mg *eow* | 113 | 10,6 | 20,5 | 33,7 | 1,83 | No/yes |  | ACR 20 | 26 weeks |
|  | I_2_: Ada 40mg *weekly* | 103 | 11,9 | 19,3 | 33,8 | 1,84 | No/yes |  |  |  |
|  | C_1_: Placebo | 110 | 11,6 | 19,8 | 35,5 | 1,88 | No/yes |  |  |  |
| **Weinblatt 2003^82^** | I_1_: Ada 40mg + MTX | 67 | 12,2 | 17,3 | 28,0 | 1,55 | Yes | 16,4 | ACR 20 24wk | 24 weeks |
|  | I_2_: Ada 80mg + MTX | 73 | 12,8 | 17,0 | 30,3 | 1,55 | Yes | 17,2 |  |  |
|  | C_1_: Placebo + MTX | 62 | 11,1 | 16,9 | 28,7 | 1,64 | Yes | 16,5 |  |  |
|  | *Ada every other week* |  |  |  |  |  |  |  |  |  |
| **Emery 2009^83^** | I_1_: Gol 100mg+placebo | 159 | 4.1 | 12 | 24,5 | 1,7 | No |  | ACR 50 24wk | 52 weeks |
|  | I_2_: Gol 50mg+MTX | 159 | 3.5 | 13 | 26 | 1,5 | No | 19,2 |  |  |
|  | I_3_: Gol 100mg+MTX | 159 | 3.6 |  | 14 | 26 | 1,6 | No |  |  |
|  | C_1_: MTX + placebo | 160 | 2.9 | 11 | 25,5 | 1,5 | No | 19,1 |  |  |
|  | *Gol every 4 weeks* |  |  |  |  |  |  | (wk 23) |  |  |
| **Kay 2008^84^** | I_1_: Gol 50mg+MTX (eow) | 35 | 8,2 | 14 | 28 | 1,7 | Yes | ≥10 | ACR 20 16wk | 52 weeks |
|  | I_2_: Gol 50mg+MTX (e4w) | 34 | 8,2 | 14 | 28 | 1,6 | Yes | ≥10 |  |  |
|  | I_3_: Gol 100mg+MTX (eow) | 34 | 6,3 | 20 | 32 | 1,8 | Yes | ≥10 |  |  |
|  | I_4_: Gol 100mg+MTX (e4w) | 34 | 9,0 | 14 | 22 | 1,3 | Yes | ≥10 |  |  |
|  | C_1_: MTX + placebo | 35 | 5,6 | 13 | 22 | 1,3 | Yes | ≥10 |  |  |
| **Keystone 2009^85^** | I_1_: Gol 100mg + placebo | 133 | 5,9 | 11 | 22 | 1,38 | Yes | 15 | ACR 20 wk 14 | 52 weeks |
|  | I_2_: Gol 50mg + MTX | 89 | 4,5 | 13 | 26 | 1,38 | Yes | 15 |  |  |
|  | I_3_: Gol 100mg + MTX | 89 | 6,7 | 12 | 23 | 1,38 | Yes | 15 |  |  |
|  | C_1_: MTX + placebo | 133 | 6,5 | 12 | 21 | 1,25 | Yes | 15 |  |  |
|  | *Gol every 4 weeks* |  |  |  |  |  |  |  |  |  |
| **Fleischmann 2009^86^** | I_1_: Cer 400mg | 111 | 8,7 | 21,2 | 29,6 | 1,4 | No/yes |  | ACR 20 24wk | 24 weeks |
|  | C_1_: Placebo | 109 | 10,4 | 19,9 | 28,3 | 1,6 | No/yes |  |  |  |
|  | *Cer every 4 weeks* |  |  |  |  |  |  |  |  |  |
| **Keystone 2008^87^** | I_1_: Cer 200mg + MTX | 393 | 6,1 | 21,7 | 30,8 | 1,7 | Yes | 13,6^2^ | ACR 20 wk 24 | 52 weeks |
|  | I_2_: Cer 400mg + MTX | 390 | 6,2 | 21,5 | 31,1 | 1,7 | Yes | 13,6^2^ |  |  |
|  | C_1_: MTX + placebo | 199 | 6,2 | 21,2 | 29,8 | 1,7 | Yes | 13,4^2^ |  |  |
| **Smolen 2009^88^** | I_1_: Cer 200mg + MTX | 246 | 6,1 | 20,5 | 30,1 | 1,6 | Yes | 12,5^2^ | ACR 20 24wk | 24 weeks |
|  | I_2_: Cer 400mg + MTX | 246 | 6,5 | 21,0 | 30,0 | 1,6 | Yes | 12,6^2^ |  |  |
|  | C_1_: Placebo + MTX | 127 | 5,6 | 21,9 | 30,4 | 1,6 | Yes | 12,2^2^ |  |  |
|  | *Cer every other week* |  |  |  |  |  |  |  |  |  |
