# Supplementary material for: Systematic Review and Meta-Analysis of the Efficacy and Safety of Existing TNF Blocking Agents in Treatment of Rheumatoid Arthritis
Source: PLoS One. 2012 Jan 17;7(1):e30275. doi: 10.1371/journal.pone.0030275 (PMC3260264; doi:10.1371/journal.pone.0030275)
Supplement: Table S3 — PRISMA Checklist of items to include when reporting a systematic review or meta-analysis. (DOCX) [file pone.0030275.s003.docx]

| Section/topic | # | Checklist item | Reported on page # |
| --- | --- | --- | --- |
| **TITLE** | | | |
| Title | 1 | “Systematic review and meta-analysis of the efficacy and safety of existing tnf blocking agents in treatment of rheumatoid arthritis” | 1 |
| **ABSTRACT** | | | |
| Structured summary | 2 | **Background and objectives***:* Five tumour necrosis factor (TNF)-blockers (infliximab, etanercept, adalimumab, certolizumab pegol and golimumab) are available for treatment of rheumatoid arthritis. Only few clinical trials compare one TNF-blocker to another. Hence, a systematic review is required to indirectly compare the substances. The aim of our study is to estimate the efficacy and the safety of TNF-blockers in the treatment of rheumatoid arthritis (RA) and indirectly compare all five currently available blockers by combining the results from included randomized clinical trials (RCT).  **Methods:** A systematic literature review was conducted using databases including: MEDLINE, SCOPUS (including EMBASE), Cochrane library and electronic search alerts. Only articles reporting double-blind RCTs of TNF-blockers *vs.* placebo, with or without concomitant methotrexate (MTX), in treatment of RA were selected. Data collected were information of patients, interventions, controls, outcomes, study methods and eventual sources of bias.  **Results:** Forty one articles reporting on 26 RCTs were included in the systematic review and meta-analysis. Five RCTs studied infliximab, seven etanercept, eight adalimumab, three golimumab and three certolizumab. TNF-blockers were more efficacious than placebo at all time points but were comparable to MTX. TNF-blocker and MTX combination was superior to either MTX or TNF-blocker alone. Increasing doses did not improve the efficacy. TNF-blockers were relatively safe compared to either MTX or placebo.  **Conclusions:** No single substance clearly rose above others in efficacy, but the results of the safety analyses suggest that etanercept might be the safest alternative. Interestingly, MTX performs nearly identically considering both efficacy and safety aspects with a margin of costs. | 2 |
| **INTRODUCTION** | | | |
| Rationale | 3 | “Previous systematic reviews and meta-analyses have studied the subject in various settings and comparisons [6–14]. These studies concluded that while TNF-blockers are efficacious but it may still be beneficial to use them in combination therapies. Only few differences in efficacy and safety between individual substances were discovered. However, more randomized clinical trials have been published lately with additional data available to systematic reviews and most importantly, two new substances, certolizumab and golimumab, have been introduced to clinical use.  ” | 3 |
| Objectives | 4 | “The purpose of this systematic review and meta-analysis is to study the efficacy and safety of all five currently available TNF-blockers in the treatment of RA compared to either methotrexate (MTX) and placebo or placebo alone and to perform an indirect comparison between individual substances in different drug combinations and doses and at different time points. We test the assumption that it is more efficacious and comparatively safer to use MTX in combination with a TNF-blocker in the treatment of RA compared to TNF-blocker monotherapy. We study if high doses of TNF-blockers differ from regular doses in efficacy and safety. Primary efficacy endpoint is the risk ratio between intervention and control group in American College of Rheumatology (ACR) 50% improvement at 6 months [15,16]. Secondary efficacy endpoints include risk ratios in ACR 20%, 50% and 70% improvements at 3, 6 and 12 months in several comparisons. Primary safety endpoint is the risk ratio between intervention and control group in the number of discontinuations due to adverse events. Secondary safety endpoints include risk ratios in the number of adverse events, serious adverse events, infections, serious infections and injection site reactions.  ” | 3 |
| **METHODS** | | | |
| Protocol and registration | 5 | Protocol has not been published online nor registered |  |
| Eligibility criteria | 6 | “According to inclusion criteria patients had to be at least 16 years of age; be diagnosed with RA using ACR 1987 criteria; and be randomized either to intervention or control group. Studies were to have one (or more) of the TNF-blockers as intervention and either placebo or combination of placebo and methotrexate as control. The TNF-blocker had to be delivered through the same route as the commercial drug and be within the dose range recommended for the commercially available products. Efficacy was measured in terms of ACR 20 %, 50 % and 70 % improvements and thus, at least one of these had to be reported at some time point. Information regarding safety had to be reported. Previously published systematic reviews were searched for, but excluded from the systematic review due to the inclusion criteria.  “ | 3 |
| Information sources | 7 | “References from (Ovid^®^) Medline, Cochrane library (Cochrane Central register of Controlled Trials, Cochrane Database for Systematic Reviews, Health Technology Assessment, Database of Abstracts of Reviews of Effects, NHS Economic Evaluation, Cochrane Methodology Register), SCOPUS (including Embase), ISI web of knowledge and several other databases were extracted and imported to reference management software (RefWorks). Clinical trial register (clinicaltrials.gov) was hand searched for unpublished trials. “ | 4 |
| Search | 8 | “Search strategy was designed and performed by a librarian by our request. We used the search terms rheumatoid arthritis, anti-TNF, infliximab, etanercept, adalimumab, golimumab, certolizumab, randomized clinical trials and systematic review. Variations in spelling were taken into account.  There were no restrictions on study language. For search strategy, see table S1.  .” | 4 |
| Study selection | 9 | “References were evaluated by two individual investigators (KA, LV) using pre-defined inclusion and exclusion criteria. Decision for inclusion was made on consensus. A third investigator (YTK) made the final decision in case of disagreement. Evaluation was based on title and abstract whenever available. Full text articles from potentially relevant references were obtained in electronic or printed format and re-evaluated for inclusion by the same investigators as before.  ” | 4 |
| Data collection process | 10 | “Data on study design, patient status and background, efficacy and safety were extracted from the publications using an Excel data extraction form by two independent researchers (KA, LV)..” | 4 |
| Data items | 11 | “…study design, patient status and background, efficacy and safety…” | 4 |
| Risk of bias in individual studies | 12 | “As instructed in the Cochrane handbook for systematic reviews of interventions, the investigators performed an evaluation of bias rather than of methodological quality. Studies included were evaluated for an eventual bias using methods described in the Cochrane handbook. The study was to be considered “possibly biased” in case a possible source of bias was found in any of the seven dimensions evaluated. The following dimensions were considered in the bias assessment tool: Allocation sequence generation, allocation concealment, blinding of participants, personnel and outcome, incomplete outcome data, selective outcome reporting and other sources of bias.  “ | 4 |
| Summary measures | 13 | “Primary efficacy endpoint is the risk ratio between intervention and control group in American College of Rheumatology (ACR) 50% improvement at 6 months [15,16]. Secondary efficacy endpoints include risk ratios in ACR 20%, 50% and 70% improvements at 3, 6 and 12 months in several comparisons. Primary safety endpoint is the risk ratio between intervention and control group in the number of discontinuations due to adverse events. Secondary safety endpoints include risk ratios in the number of adverse events, serious adverse events, infections, serious infections and injection site reactions. ” | 3 |
| Synthesis of results | 14 | “Data were analyzed using the intention to treat results from the included studies. Meta-analyses were performed using Cochrane Collaboration Review Manager 5.0 software. Sensitivity analyses were employed to account for the possible bias. In some settings several time points were combined to increase the power. Efficacy and safety were analyzed using dichotomous data to obtain risk ratios. Dichotomous efficacy data included ACR 20 %, 50 % and 70 % improvements whereas dichotomous safety data was composed of the proportion of patients who experienced an adverse outcome or discontinued the treatment due to adverse events. The efficacy and safety of TNF-blockers was analyzed in six different main comparisons. Random effects model was used to account for the diversity of the studies. Heterogeneity was evaluated via subgroup analysis using Chi square and I^2^-statistics. | 4 |
| Risk of bias across studies | 15 | Risk of bias across studies was not formally assessed |  |
| Additional analyses | 16 | “Sensitivity analyses were employed to account for the possible bias.”, “To investigate the possible effect of patients’ baseline disease activity on efficacy, two additional analyses were performed.”, In addition, several subgroup analyses were performed. | 4-6 |
| RESULTS | | | |
| Study selection | 17 | After removing duplicate entries, 3841 references were evaluated for inclusion based on title and/or abstract. Seventy six potentially relevant references were included in the next stage, where the publication was to be re-evaluated based on full text (figure 1). Full text was unavailable for 12 studies most of which were conference abstracts identified from ISI Web of Knowledge [18–29]. Patients, interventions, controls, outcomes or design of the studies did not meet the inclusion criteria of the systematic review in 17 publications [30–46]. Five review articles, one letter to the editor [47] and one erratum [48] were excluded. Several of the remaining 41 publications were reporting on a single study and were thus merged into one (table S2). Publications included in the systematic review and meta-analysis are listed in the bibliography with numbers 48-88. From the 26 clinical trials included in the systematic review, 8 used adalimumab, 7 etanercept , 5 infliximab, 3 golimumab and 3 certolizumab for intervention. The included trials have 9862 patients of which 6780 and 3082 were in intervention and control groups, respectively (table S2). | 5 |
| Study characteristics | 18 | Study characteristics are described in table S2. | 13-14 |
| Risk of bias within studies | 19 | “A potential source of bias was discovered in five trials included in the systematic review (table 1). In many clinical trials there was an early escape route for patients with insufficient treatment response to avoid rapid disease progression. In some studies this was implemented by considering all patients failing to meet a pre-defined treatment response criteria (e.g. ACR 20 % improvement) as “non-responders” before the actual efficacy assessment. While this may be for the best interest of the study subjects, it may introduce a bias to the evaluation of the efficacy results. Another bias was caused by switching the control group to active medication.  ” | 5 |
| Results of individual studies | 20 | The results of the individual studies are shown in figures 2 and 3. |  |
| Synthesis of results | 21 | “The primary efficacy endpoint of our study was the risk ratio of 50 % improvements in the ACR-treatment response criteria at six months between intervention and control group. Fourteen trials were included and of them 2 used infliximab, 2 etanercept, 5 adalimumab, 2 golimumab and 3 certolizumab for intervention. As a group, TNF-blockers reached a risk ratio of 4.07 (95 % CI 2.70-6.13) regarding the achievement of the efficacy endpoint compared to controls. For infliximab, etanercept, adalimumab, golimumab and certolizumab the corresponding figures were 3.08 (0.91-10.43), 8.61 (3.55-20.86), 4.34 (3.30-5.70), 1.56 (0.93-2.60) and 5.95 (3.97-8.92), respectively (figure 2)..  “Significant heterogeneity was present in the first analysis comparing any intervention to any control. Heterogeneity diminished as the comparisons were stratified into smaller comparisons.  .”  “The primary safety endpoint of the systematic review was the discontinuation of study due to adverse events. There were 25 studies with 6292 patients in the intervention and 2994 in the control group in this analysis (table 6). As a group, the TNF-blockers did not statistically significantly differ from the control (RR 1.26, CI 95% 0.93-1.71). While the patients on infliximab (3.22, 1.76-5.91), adalimumab (1.59, 1.13-2.23), and certolizumab (2.72, 1.23-6.01), had an increased risk to discontinue, the patients on etanercept (0.71, 0.54-0.92) had a decreased risk (figure 3). Patients using certolizumab had a higher risk to experience a serious adverse event than patients on etanercept with risk ratios of 2.24 (1.38-3.63) and 0.90 (0.68-1.20), respectively. Infliximab, etanercept and golimumab increased the likelihood of an injection or infusion reaction while adalimumab and certolizumab did not statistically significantly differ from the controls in this respect although their risk ratios leaned to the same direction.  In addition to the primary outcome measures, efficacy and safety were analysed in six comparisons (tables 2-6). | 5-8 |
| Risk of bias across studies | 22 | Risk of bias across studies was not formally assessed. |  |
| Additional analysis | 23 | “The sensitivity analyses based on the results of the bias assessments did not reveal any statistically significant bias on the efficacy results. Occasionally, however, the statistical significance between intervention and control groups disappeared due to reduced number of studies. In the sensitivity analyses, the estimate of the risk ratio decreased, increased or remained the same in 52%, 45% and 3% of cases, respectively. In some cases there were no clearly unbiased RCTs in a comparison, thus making it impossible to perform the sensitivity analysis..”  “. Trials with low swollen joint count and low HAQ score had risk ratios of 3.43 (CI 95% 2.03-5.78) and 3.68 (2.11-6.42), respectively, whereas trials with high swollen joint count and high HAQ score had risk ratios of 5.15 (2.72 – 9.75) and 4.64 (2.59-8.31), respectively.  ”  Additionally, several post-hoc subgroup analyses were performed.  “Patients on either infliximab or adalimumab with disease duration more than 2 years were more likely to reach ACR 20, 50 and 70 at 12 months compared to controls than patients with disease duration less than two years (table 2). ” At six months patients previously naïve to MTX are statistically significantly less likely to reach either ACR 20, 50 or 70 treatment responses compared to patients who had already been previously treated with MTX”  “In a subanalysis of trials with patients who had previously used MTX, the results were similar. In comparison to MTX, golimumab combination therapy was still inferior in ACR 20 efficacy at 6 months to certolizumab combination therapy, with risk ratios of 2.14 (1.59-2.89) and 5.08 (3.46-7.48), respectively.“At six months patients previously naïve to MTX are statistically significantly less likely to reach either ACR 20, 50 or 70 treatment responses compared to patients who had already been previously treated with MTX”.”  “Stratifying RTCs by previous exposure to MTX does not show any statistically significant differences in the treatment response to TNF-blocker monotherapy between these two groups. | 5-6 |
| DISCUSSION | | | |
| Summary of evidence | 24 | “Our systematic literature search identified 40 publications reporting 26 randomized controlled trials studying the efficacy and safety of TNF-blockers. The included trials were published 1999-2010. Five trials published used infliximab [49–59], seven etanercept [60–72], eight adalimumab [73–82], three golimumab [83–85] and three certolizumab [86–88] for the intervention. Overall, there were 6780 patients in the intervention and 3082 in the control group. The patients’ characteristics varied across the included trials with mean time since diagnosis ranging from 0.5 to 13 years, HAQ score from 1.25 to 1.88 and the number of swollen and tender joints from 11 and 14.03 to 24 and 37.2, respectively.  The results of the primary efficacy endpoint suggest that infliximab and golimumab do not statistically significantly differ from control regarding efficacy in a comparison between any combination of TNF-blocker and any control. Even though the different settings and heterogeneity among the studies could have accounted for the result, the finding still raises questions. Golimumab appears to be inferior in efficacy to etanercept, adalimumab and certolizumab even after accounting for the eventual bias. Patients in golimumab trials have lower count of swollen and tender joints as well as lower HAQ score, which may explain the results to some extent, although an ad hoc analysis on the effect of patient characteristics on efficacy showed no statistical significance. Disease duration seems to predict treatment response to adalimumab and infliximab at 12 months.  In the second and third meta-analysis the efficacy of MTX and TNF-blocker combination was found to be superior to either MTX or TNF-blocker alone, respectively. The increase in the number of discontinuations due to adverse events (RR 1.37 95% 1.01-1.87) compared to MTX alone is likely to be acceptable. Patients with previous exposure to MTX were more likely to benefit from the combination therapy compared to MTX naïve patients. Compared to monotherapy with a TNF-blocker the safety of the combination treatment was equal or even improved regarding some aspects.  The fourth meta-analysis found no statistical difference between MTX and TNF-blocker monotherapy and the fifth one confirmed that TNF-blocker monotherapy was more efficacious than placebo. The last secondary efficacy meta-analysis found little benefit from increasing the dose of TNF-blockers.  In the first safety comparison between TNF-blockers and control the risk ratios reached statistical significance only in the number of patients experiencing injection or infusion reactions. Interestingly, infliximab, adalimumab and certolizumab increased the risk of discontinuation of treatment due to adverse events, but etanercept made it less likely. Certolizumab was the only TNF-blocker which increased the likelihood of experiencing a serious adverse event. While TNF-blockers as a group increased the odds to experience an injection or infusion reaction this may not be the case with adalimumab and certolizumab.  ” | 9-10 |
| Limitations | 25 | “It could be asked, whether TNF-blocker naive and switchers should be included in the same review, because these patients could be very different. However, fifteen trials included in this systematic review stated previous TNF-blocker use as an exclusion criterion. In eight more trials it was unclear if switchers were included and only two certolizumab trials included switchers but excluded those who had had insufficient response to previous TNF-blocker treatment. However, the percentage of previous TNF-blocker users in these two trials was small (2 – 4 %) and a sensitivity analysis was performed.  While broader comparisons with larger number of trials may be more likely to reach statistically significant results (1.00 not included in the confidence interval), their validity may be questioned. Heterogeneity introduced by combining the results of trials with different settings causes random effects model to calculate wider confidence intervals than fixed effects model would do. While reducing the possibility of type I error, it may introduce a type II error. Hence, the efficacies of TNF-blockers were compared with different controls, combinations and dosages in smaller, but more homogenous comparisons.  Results of the sensitivity analyses revealed that the source of bias in the RCTs is as likely to lead to underestimation as overestimation of the risk difference between intervention and control groups. However, the homogeneity of study population, intervention, control, outcomes and study settings are likely to be more crucial to the validity of the meta-analysis. Length of exposure was not taken into account in the safety analyses, only the difference in risk ratios between intervention and control group.  Our systematic review and meta-analysis has some limitations. The authors of the included trials were not contacted to retrieve unpublished data. Many studies that lasted for one year or more only reported results at 12 months. The meta-analyses would have been more powered if the efficacy results had been reported at all time points. Selective reporting was included in the evaluation for bias, but we were unable to identify any bias here.  .  “ | 9 |
| Conclusions | 26 | “The novel TNF-blockers may offer an alternative to older substances but do not make them obsolete. On the contrary, etanercept may be the best choice when taking into account safety profiles of the TNF-blockers. Infliximab, etanercept and adalimumab have been in clinical use for years with extensive amount of post-marketing data available. More post-marketing information is needed on certolizumab and golimumab for comprehensive pharmacovigilance.  The annual medication costs of TNF-blockers are more than 10 000€ while the MTX treatment costs less than 100€ per year. Subgroup analysis in table 3 suggests that considering the high expenses of biologics, the treatment of RA could be initiated with MTX while combining TNF-blockers to ongoing treatment in patients with insufficient response to MTX. Even though safety was not compromised, it might not be cost-effective to use high doses of TNF-blockers. Given the limited resources in healthcare systems our results may help clinicians and decision makers to get most out of the expensive, but efficacious treatment.  The next step could be to analyze the efficacy and safety of not just TNF-blockers, but all biologics in a large systematic review and meta-analysis. One randomized clinical trial included in our systematic review actually compared abatacept to infliximab [55]. However, a systematic review is indicated to summarise the evidence.  ” | 11 |
| FUNDING | | | |
| Funding | 27 | “This study was support by an unlimited grant from the ORTON Orthopaedic Hospital of the ORTON Foundation and by the National PhD Graduate School in Musculoskeletal Diseases and Biomaterials.” | 11 |
